# Preparation and identification of chitosan and chitosan nanoparticles from crab byproducts

**DOI:** 10.1038/s41598-026-60387-z

**Published:** 2026-07-09

**Authors:** Abdelrahman S. Talab, Ahmed M.S. Hussein, Mohie M. Kamil, Sayed Mostafa, Gamal H. Ragab, Hala E. Ghannam, Alaa I. Khedr

**Affiliations:** 1 National Institute of Oceanography and Fisheries, NIOF, Cairo, Egypt; 2 Food Technology Department, Food Industries and Nutrition Research Institute, National Research Centre, Dokki, Cairo, 12622 Egypt

**Keywords:** Chitosan NPs, Blue crab shell, SEM, XRD, TEM, Viscosity, Biotechnology, Environmental sciences, Materials science, Nanoscience and technology

## Abstract

This study was designed to extract chitosan from blue crab shells and prepare chitosan nanoparicles via combination of chemical extraction process with mechanical ball-milling method. The size and surface charge of the NPs were evaluated in chitosan nanoparicles using dynamic light scattering (DLS). The prepared crab chitosan and chitosan nanoparicles were evaluated for their characteristics using Fourier Transform Infrared Spectroscopy (FTIR), X-ray Diffraction (XRD), Scanning Electron Microscopy coupled with Energy Dispersive X-ray analysis (SEM-EDX), Transmission Electron Microscopy (TEM) and viscosity measurements compared with commercial chitosan products. The DLS results of crab chitosan nanoparicles appeared to be the optimal chitosan nanoparicles with minimum particle size (192.74 ± 3.25 nm), zeta potential (25.15 ± 1.19 mV) smallest PDI (0.228 ± 0.011) in comparison with commercial nanoparicles had a particle size (245.47 ± 3.21 nm), zeta potential (32.24 ± 0.72 mV) smallest PDI (0.191 ± 0.055), respectively. The FTIR and XRD analyses indicated that α-chitosan mainly formed an amorphous structure along with a higher deacetylation degree. There are no diffraction peaks at 2θ ≈ 10°, showing that chitin was effectively deacetylated with a negligible content of residual chitin. Chitosan nanoparicles from crab had a particle size of about 84 nm based on Debye–Scherrer equation that is comparable to commercial nanoparticles. Crab chitosan nanoparicles has been shown to possess distinctly rough flake-like surfaces, while CS NP is compact spheres to cubes with reduced pore dimensions, as confirmed by SEM and TEM analyses. EDX with low residual mineral contents confirmed good purities of prepared materials. Rheological analysis indicated that chitosan extracted from crabs exhibited greater viscosity (31.707 to 42.849 mPa·s) than commercial chitosan (17.818 to 24.024 mPa·s), indicating a higher molecular weight and more significant intermolecular interactions. The overall chitosan yield was 11.02% of the crab shell dry weight. The findings suggest that blue crab shell waste is a value-added product. .

## Introduction

Chitosan (CS) is one of the most important naturally derived polymers due to its biodegradability, biocompatibility, low toxicity, and versatile physicochemical properties^1,2^. It is generated by the deacetylation of chitin, which is one of the most widespread natural polysaccharides on Earth after cellulose^3^. Chitin is abundant in nature, forming a significant structural part of the shells of crustaceans, fungal cell walls, the exoskeletons of insects and some marine organisms^4^^[,5,6^. Moreover, chitin has been recognized in fungi, algae, velvet worms, and protozoa^7^. In addition, chitin is present in the shells of Arthropoda, including insects and worms. The skeleton is a robust and rigid structure that functions as a support system for the body and a defense against predators^8^. The chitin-protein interaction produces an enduring filamentous structure^9^. The chitin content of crab shells ranges from 6% to 72%, contingent upon the species^10^. This notable disparity may be ascribed to various elements, encompassing the origin of the biomass, the segment of the biomass assessed for chitin analysis, and the analytical method employed^11^. In addition, the chitin content varies significantly according to its developmental stage. Wasp larvae possess a reduced chitin concentration compared to pupae and adults^12^.

Statistics demonstrates that a billion kilogram of seashells from mollusks are generated annually worldwide^13^. Globally, there are around 4,500 species of crab. However, only a limited number of these species have been utilized for chitosan production and subjected to physicochemical analysis^14^. Crustacean shell wastes, which are a byproduct of the seafood processing industries, represent one of the most commercially valuable and sustainable raw materials for chitosan production out of these sources^15^. Excessive accumulation of shellfish processing wastes has constituted an environmental menace in numerous coastal habitats, resulting in bad odor, microbial contamination and disposal problems^16^. Therefore, the valorization of crustacean biowaste into valuable biomaterials has attracted intensive scientific and industrial interests. In this study, blue crab (*Callinectes sapidus*) shells were chosen as an abundant crustacean biowaste product produced from seafood processing industries and local fish markets. Moreover, blue crab shells with a high content of chitin and low availability cost are the best choice for renewable raw materials from industrial production of chitosan^17,18^. In this sense, shells of blue crab (*Callinectes sapidus*) represent a rich and yet underexploited source of chitin-enriched biomass in particular along the Egyptian Mediterranean coast^19^. Massive amounts of blue crab shell wastes are discarded every year although they contain high contents of chitin and represent raw materials with economic value^20^. Thus, recycling these wastes into chitosan is an eco-friendly solution for waste treatment and recovery.

Chitosan is traditionally prepared by sequential demineralization, deproteinization and deacetylation treatments with acid–alkali^9^. Several alternative extraction methods, including enzymatic, ultrasound-assisted, ionic liquid and deep eutectic solvent ones have been reported; however, due to their easy implementation, efficiency and economic viability chemical extraction is still the most widely used industrial process^21^^[,22,23^. Extraction conditions strongly affect the physicochemical properties, such as degree of deacetylation (DD), crystallinity, viscosity, molecular weight and surface morphology of produced chitosan. These features play a direct role in its industrial, biomedical, and environmental relevance^24^.

Chitosan nanoparticles (CS NPs) have gained much interest in the past years because of their superior surface area, excellent biocompatibility, minimal toxicity, adsorption capacity, colloidal stability and functional reactivity compared to bulk chitosan^12^, rendering it a versatile material applicable in various fields such as biomedicine, food, cosmetics, dietary supplements, chemistry, materials science, and packaging^25^. A number of techniques have been introduced for the preparation of CS NP, including ionic gelation, emulsion crosslinking, solvent precipitation and mechanical milling^26^. It also gives rise to sustainable processing, and a simple but scalable mechanical milling method provides good solvent-minimized route for size reduction of any desired nanoscale material without chemical crosslinkers that can change the inherent functional groups of chitosan^27^. Moreover, stabilizing agents (e.g., polyethylene glycol, PEG) can minimize particle agglomeration and improve dispersion throughout milling^28^. PEG 400 (polyethylene glycol, MW ~ 400) was employed as a surfactant/dispersant, a steric stabilizer, lubricant, and nanoparticle protector, enabling effective mechanical size reduction into the nanometer range without aggregation or polymer degradation. PEG coats the chitosan particles, and reduces friction between: milling balls, chitosan particles, and the milling jar and reducing surface energy, thus it prevents local overheating, avoids agglomeration or polymer melting, and allows more uniform size reduction. PEG also improves hydrophilicity and colloidal stability, keeping the milled particles suspended rather than clumping together. In addition, PEG is ideal because it is non-toxic, w soluble, non-ionic, non-reactive with chitosan’s amino groups, and easily removable after milling. It is used in polymer-to-polymer proportions, typically on the order of a few to several tens of percent relative to chitosan mass, depending on PEG molecular weight and target nanoparticle size^29^.

While an extensive number of studies has been published around chitosan extracted from crustaceans, characterization of chitosan produced from blue crab shell waste is not available yet. Most previous studies had focused either on extraction yield or chitin content, with lack of structural, morphological and physicochemical characterization^30^. In this context, the purpose of this study was to prepare chitosan by conventional physical-acid alkaline based extraction method from shell waste of blue crab (*C. sapidus*) as well as to synthesize nanoparticles through mechanical ball milling. Fourier-transform infrared spectroscopy (FTIR), X-ray diffraction (XRD), scanning electron microscopy coupled with energy-dispersive X-ray spectroscopy (SEM–EDX), transmission electron microscopy (TEM) and viscosity were used extensively in the characterization of the synthesized chitosan and chitosan nanoparticles.

## Materials and methods

### Chemicals

Sodium Hydroxide (NaOH), Hydrochloric Acid (HCl), Polyethylene Glycol, Acetic Acid. The commercial chitosan with molecular weights (MW) of ~ 190 kDa (Sigma Aldrich, St. Louis, MO, USA) was purchased from Sigma Aldrich. The DD values reported in the present study were independently estimated from FTIR spectral analysis and therefore may differ slightly from the supplier specification.

### Synthesis

The crab exoskeletons (*Callinectes sapidus*) were obtained from a local market in Egypt. The production of CS from crab shells was conducted according to the procedure established by^30,31^. The crab exoskeleton waste included mixed shell parts of pooled *Callinectes sapidus*, such as carapace, claws and walking legs plus attached external shell fragments. The exoskeleton parts were rinsed with distilled water several times to remove salts, blood, and surface organics. Furthermore, washing in hot deionized waster was processed for 5 to 10 min to improve the removal of loosely bound organics. Afterwards, “Extracts of washed crab shells were oven-dried at 60 °C until constant weight to evaporate remaining moisture, then pulverized into fine powder utilizing a grinder and mechanically milled and sieved into a fine powder that has an approximate average particle size of 37 µm”.

Afterwards, approximately 250 g of fine crab exoskeletons were rinsed with 4 N HCl (1:14 w/v) at 30 °C for 36 h. This step is performed to eliminate inorganic components predominantly calcium carbonate, from the crab exoskeleton matrix. The 4 N HCl solution at a solid/liquid ratio of 1:14 (w/v) was used to convert mineral phases into soluble salts, provide excellent acid availability for calcium carbonate and other mineral components due to the crab exoskeleton; thereby liberating the organic chitin framework. After completion of the demineralization reaction, the solid fraction was separated from the acid liquor and subjected to repeated washing with distilled water to remove residual acid and dissolved mineral species. Washing was continued until the pH of the filtrate attained 6.5–7. In addition, confirm minimal residual chloride (e.g., absence of precipitate upon adding a drop of AgNO₃ to filtrate). This neutralization step is essential to prevent acid carry-over that could interfere with subsequent deproteinization or cause degradation of the biopolymer. The demineralized shells were subsequently dried to constant mass to remove physically bound moisture and to ensure consistent material composition prior to further chemical processing. The demineralized shells were further digested with a 5% NaOH solution (1:12 w/v) at 90 °C for 24 h. This deproteinization step was processed to remove residual proteins bound to the chitin matrix through hydrogen bonding, electrostatic interactions, and covalent cross-links. 1:12 (w/v) NaOH had been used to avoid excessive alkaline degradation of the chitin polymer backbone during deproteinization.

**Fig. 1:**
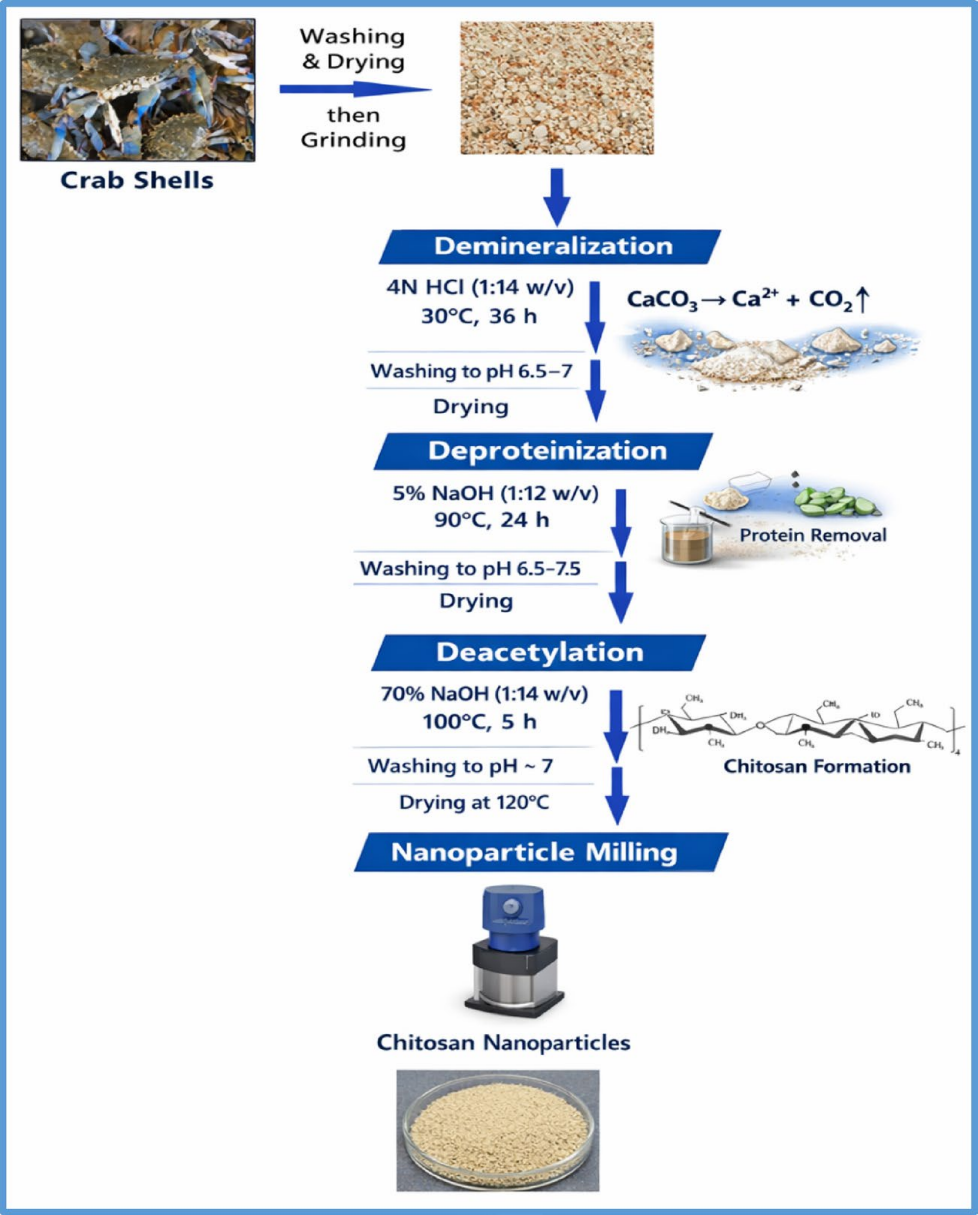
Schematic diagram of chitosan extraction, CS, CS NPs biosynthesis from blue *C. sapidus* crab exoskeletons.

Following alkaline digestion, the solid chitin phase was separated from the liquid containing dissolved proteins and peptides. The recovered solids were then thoroughly washed with distilled water to eliminate residual alkali and solubilized organic matter. Washing was continued until the pH of the washings reached a near-neutral range (approximately 6.5–7.5), which was used as an indicator of effective removal of excess hydroxide ions and protein degradation products. This step is critical to prevent alkaline degradation of the polymer and to ensure chemical stability in subsequent processing. The deproteinized material was subsequently dried to constant weight to remove physically adsorbed moisture and to obtain a chemically stable chitin-rich solid. The chitin acquired from the aforementioned procedure was desiccated, pulverized, and sieved, thereafter undergoing deacetylation in 70% NaOH (1:14 w/v) for 5 h at 100°C^32^. Deacetylation was conducted to convert chitin into chitosan by removing acetyl groups from the N-acetyl-D-glucosamine units of the polymer. It was well known that concentrated alkali under an elevated temperature condition can turnover chitin into high-deacetylation degree chitosan efficiently, therefore, the 70% NaOH solution with the mass ratio of 1:14 (w/v) was selected for deacetylation. As deacetylation proceeds, the polymer undergoes a transition from a highly crystalline, insoluble biopolymer (chitin) to a more amorphous and chemically reactive polymer (chitosan). After completion of the deacetylation reaction, the solid CS was separated from the alkaline medium and subjected to repeated washing with distilled water to remove residual alkali and other soluble by-products. Washing was continued until the pH of the washings reached 6.5–7.5. Ensuring chemical stability and preventing alkaline degradation of the polymer. The purified chitosan was subsequently dehydrated in a furnace at 120 °C for 24 h to constant mass, and afterwards pulverized, yielding a stable biopolymer suitable for nanoparticle preparation^33^, Fig. 1. The chitosan yield was calculated from the crab shells (dry weight) using an analytical balance according to Eq. 1.

1 $$\:\boldsymbol{Y}\boldsymbol{i}\boldsymbol{e}\boldsymbol{l}\boldsymbol{d}\:\mathrm{\%}=\:\frac{\boldsymbol{W}\boldsymbol{e}\boldsymbol{i}\boldsymbol{g}\boldsymbol{h}\boldsymbol{t}\:\boldsymbol{o}\boldsymbol{f}\:\boldsymbol{C}\boldsymbol{h}\boldsymbol{i}\boldsymbol{t}\boldsymbol{o}\boldsymbol{s}\boldsymbol{a}\boldsymbol{n}\:\left(\boldsymbol{g}\right)}{\boldsymbol{W}\boldsymbol{e}\boldsymbol{i}\boldsymbol{g}\boldsymbol{h}\boldsymbol{t}\:\boldsymbol{o}\boldsymbol{f}\:\boldsymbol{C}\boldsymbol{r}\boldsymbol{a}\boldsymbol{b}\:\boldsymbol{s}\boldsymbol{h}\boldsymbol{e}\boldsymbol{l}\boldsymbol{l}\boldsymbol{s}\:\left(\boldsymbol{g}\right)}\:\times\:100$$

The ball milling process (Model: PQ-N2 Planetary Ball Mill) was employed to reduce particle size and prevent particle agglomeration from getting the chitosan nanoparticles (CS NPs)^34^. According to Joni et al.^33^, crab CS NPs were prepared using a Gear Drive 4-station planetary ball mill, 220 v) in the following manner: Twenty-five grams of CS powder were subjected to ball milling in a 200 ml agate vessel containing 130 zirconia beads, varying in diameter from 0.5 to 1.5 mm (75 beads of 0.5 mm, 30 beads of 1.0 mm, and 25 beads of 1.5 mm). The milling process was conducted at 4000 rpm using a high-energy planetary ball mill.

### Milling procedure

The dried CS powder was ground into a fine powder for 90 min. The fine powder was sieved with a mesh size of 400 to achieve a more consistent micron size (about 37 μm) and was exposed to the bead milling process following the method of^33^. The bead mill comprises a 200 cc jar, a pump, and a mixing tank. The container was filled with beads to 70% of its maximum capacity. The slurry was created by combining CS powder with distilled water. The CS mixture was agitated with a magnetic stirrer for 30 min before bead milling. The PEG 400 (polyethylene glycol) dispersion agent was incorporated following a 15-minute mixing period. The PEG was utilized as a surfactant/dispersant, a steric stabilizer, lubricant, and nanoparticle protector in the milling process. The CS slurry was introduced into the vessel containing zirconia beads, with the impeller operating at a speed of 4000 rpm. The fragments were stirred in the vessel’s lower section (dispersing area) to disintegrate the aggregate and prevent the aggregation of CS NPs in the suspension. Subsequent to dispersion, the suspension was moved from the dispersing zone to the separation area, where centrifugal force was employed to isolate the zirconia beads from the particle suspension. The CS particle suspension was thereafter returned to the dispersing portion. To maintain a constant system temperature of 25 °C, the vessel was insulated by a water jacket system and was entirely sealed. CS particles suspended in water, with a weight fraction of 0.1 wt%, were milled for 90 min, therefore accompanied by the inclusion of a 1% (v/v) acetic acid solution to enhance the positive surface charge, establishing excellent technical conditions for bead milling. Then the nanoparticles obtained were repeatedly washed with distilled water before freeze-drying, such that residual PEG could be reduced to a minimum. Nevertheless, the remaining quantity of PEG was not evaluated and probably only represents a small contribution to some physicochemical properties of the primary end-products. Following the milling procedure, CS underwent freeze-drying (LABCONCO, Kansas City, USA) at 50 °C and 0.014 mbar for 2 days to achieve a moisture level of 4%.

### Characterization

The particles size, PDI, and zeta potential of chitosan nanoparticles were done at 25 ± 0.5 °C by dynamic light scattering (DLS) technique using a Zetasizer 3000, Malvern Instruments, Malvern, UK). The materials were evaluated using Fourier transform infrared (FTIR) spectroscopy to ascertain the surface functional groups. Two milligrams of nanoparticles were mixed with 0.2 g of potassium bromide (FT-IR grade) and compressed into a granule. The sample granule was positioned in the sample container, and FT-IR spectra were obtained using an FT-IR instrument (NicoletTM iSTM5, Thermo Scientific, Madison, WI, USA) within the range of 4000 to 400 cm^− 1^ at a resolution of 4 cm^−1 35^. While FTIR Spectroscopy is an established and rapid method for the quantification of deacetylation, values obtained using this technique can diverge from those calculated by more quantitative methods like potentiometric titration, elemental analysis or nuclear magnetic resonance spectroscopy. The deacetylation degree (DD) was computed from the FTIR spectra results as in Eq. 2:

2 $$\:\boldsymbol{D}\boldsymbol{D}\:\mathrm{\%}=100-[\frac{{(\boldsymbol{A}}_{1629}-{\boldsymbol{A}}_{3450})}{1.33}\:\times\:100]$$

Where A_1629.85_ cm^1^: the absorbance intensity at 1629.85 cm^1^, corresponding to C = O of acetamide as evidence of the chitin residual. A_3450_: the absorbance intensity at 3450.65 cm^1^, corresponding to NH_2_ of the CS product. The 1.33 constant value was used because this is consistent with previously published FTIR calibration methods for analyzing deacetylation of chitosan^35^.

The nanoparticle size and crystalline structure were assessed by X-ray diffraction (XRD) investigation. This was conducted with a Philips PW-371 diffractometer operating at 40 kV and 30 mA with Cu Kα radiation (λ = 1.54056 Å). The materials have been processed across a 2θ angle range of 20 to 80 degrees at a rate of 5º/min. XRD is a quick scientific technique primarily applied for recognizing the mineral phases and can yield data on unit cell size. The examined sample was meticulously pulverized, and the mean bulk composition was ascertained. The crystalline size was ascertained utilizing Debye-Scherrer’s equation (Eq. 3).

3 $$\:\boldsymbol{D}=\frac{0.94\times\:\boldsymbol{\lambda\:}}{\boldsymbol{\beta\:}\mathbf{Cos}\boldsymbol{\theta\:}}$$

where λ represents the wavelength (Cu-Kα), B denotes the full-width half-maximum (FWHM), and Ɵ signifies the diffraction angle^36^.

The surface structure of the extracted crab CS and CS NPs and their commercial analogues were analyzed using scanning electron microscopy (SEM) (Hitachi S-4500) at a resolution of 5,000 and an accelerating voltage of 20 kV, in accordance with the procedures of^37^. The surface morphology and elemental analysis were performed with Scanning Electron Microscopy-Energy Dispersive X-ray (SEM-EDAX) (JEOL JEM-2100, Tokyo, USA) at 5 kV. For viscosity measurements, the rheological description of the CS solutions (0.1% w/v acetic acid) was conducted utilizing an Anton Paar RhoLabQC (CC27). The temperature was maintained at 25.0± 1 °C. The shear rate was adjusted from 1 to 100 s^− 1^ with a measurement length of 100 s to provide stable readings^38^ The morphology of the chitosan and its nanoparticles were analyzed according to Ottaviani et al.^39^ by transmission electron microscopy TEM (Transmission Electron Microscopy) HR-TEM (JEOL, JEM-2100, Tokyo, Japan). The present work did not include the direct molecular weight determination by gel permeation chromatography (GPC), Mark–Houwink relation, or size exclusion chromatography (SEC) intrinsic viscosity calculation. That is why viscosity measurements were only used as indirect probes of polymer chain dynamics and solution rheology.

### Data analysis methods

The analysis was conducted on four types of chitosan samples with three replications (*n* = 3) and presented as average values and standard deviations as basic statistical parameters.

## Results and discussion

CS is derived from the deacetylation of chitin, and it is designated as chitosan if the deacetylation is above 50% Nqoro et al.^40^. CS is a fundamental copolymer formed through the deacetylation of chitin, leading to the emergence of amine groups that confer cationic properties to CS^41^. The present work employed acid digestion (4 N HCl) for demineralization of salt ions from the crab exoskeleton with the ratio of exoskeleton to HCl of 1:14 (w/v) at ambient temperature (approximately 30 °C) for 36 h. HCl can eliminate minerals such as CaCO_3_ and CaCl_2_ from the crab exoskeletons through an ion exchange mechanism and produce chitin. Chitin exhibits a strong association with proteins^42^. Furthermore, deproteinization is essential in the extraction process. The deproteinization process utilized a 5% NaOH solution at a concentration ratio of 1:12 (w/v) at 90 °C for 24 h. The alkali reaction during deproteinization entails the saponification of lipids and the solubilization of proteins.

Gîjiu et al.^43^ maximized processing conditions of chitin from *Liocarcinus holsatus* and *Pachygrapus golton*, primarily located in northern Romania, demonstrating that 5% NaOH was optimal for deproteinization at 75 °C. Furthermore, the most crucial stage is the deacetylation of chitin derived from the crab. This procedure utilized alkali hydrolysis using a 70% NaOH solution, maintaining a chitin to NaOH concentration ratio of 1:14 (w/v) at a deacetylation temperature of 100 °C for a duration of 5 h. Processing parameters, including temperature, deacetylation duration, chitin-to-alkali dosage proportion, and the particle size distribution of chitin can regulate the deacetylation degree (DD) of the resultant CS. The deacetylation temperature may elevate the DD value, although it may reduce the extent of polymerization and/or molecular weight of the resultant CS^44^.

### Chitosan yield

The chitosan yield was estimated from the crab shells based upon the dry weight measurements using Eq. 1. This study generated a good chitosan yield of about 11.02 ± 0.55%. Similar results were reported by Özbay et al.^45^ reported that the chitosan content of *C. sapidus* was generated as 7.55%. While Kaya et al.^46^ found that, the chitosan yield of *C. sapidus* was reported as 9.2%. On the other hand, Talab et al.^34^ found a higher chitosan yield of 16.33% from crab compared with 15.35% CS yield from shrimp. Also, Pratiwi et al.^47^ obtained the highest yield value from swimming crab at 12.4%±0.9, followed by shrimp (8.7%±0.5) and crab (4.0%±0.5). Longo et al., 2025^48^ reported that, the average chitosan yield of *C. sapidus* was 10.71 ± 0.48% before purification and 7.93 ± 0.34% after purification.

### Characterization

#### Particle size and zeta potential

The results of particle size, zeta potential, and polydispersity index (PDI) for both commercial and crab chitosan nanoparticles (Cs NPs) were reported as follows: the DLS results of crab CsNPs appeared to be the optimal CS-NPs with minimum particle size (192.74 ± 3.25 nm), zeta potential (25.15 ± 1.19 mV) smallest PDI (0.228 ± 0.011) in comparison with commercial CsNPs had a particle size (245.47 ± 3.21 nm), zeta potential (32.24 ± 0.72 mV) smallest PDI (0.191 ± 0.055), respectively. Du et al.^49^ proposed that the NH_2_ groups present in the chitosan polymer chains may be connected to the particle size. The larger particle size of chitosan nanoparticles may have been caused by a stronger protonation of the NH_2_ moiety, which increased molecular repulsion and caused the chitosan polymer chain to stretch. However, because of a decreased chain entanglement propensity, a lower MW might not result in smaller nanoparticles. Compared to lower MW chitosan, higher MW chitosan chains might be able to entangle with one another to form more compact particles, while the highest zeta potential value, with a value greater than 30 mV, indicating it was the most stable sample among all samples studied^50^. In case of nanoparticles, a PDI below 0.3 is desired, since values higher than 0.3 indicate low uniformity, being an indication of aggregation^51^.

#### FTIR analysis

**Fig. 2:**
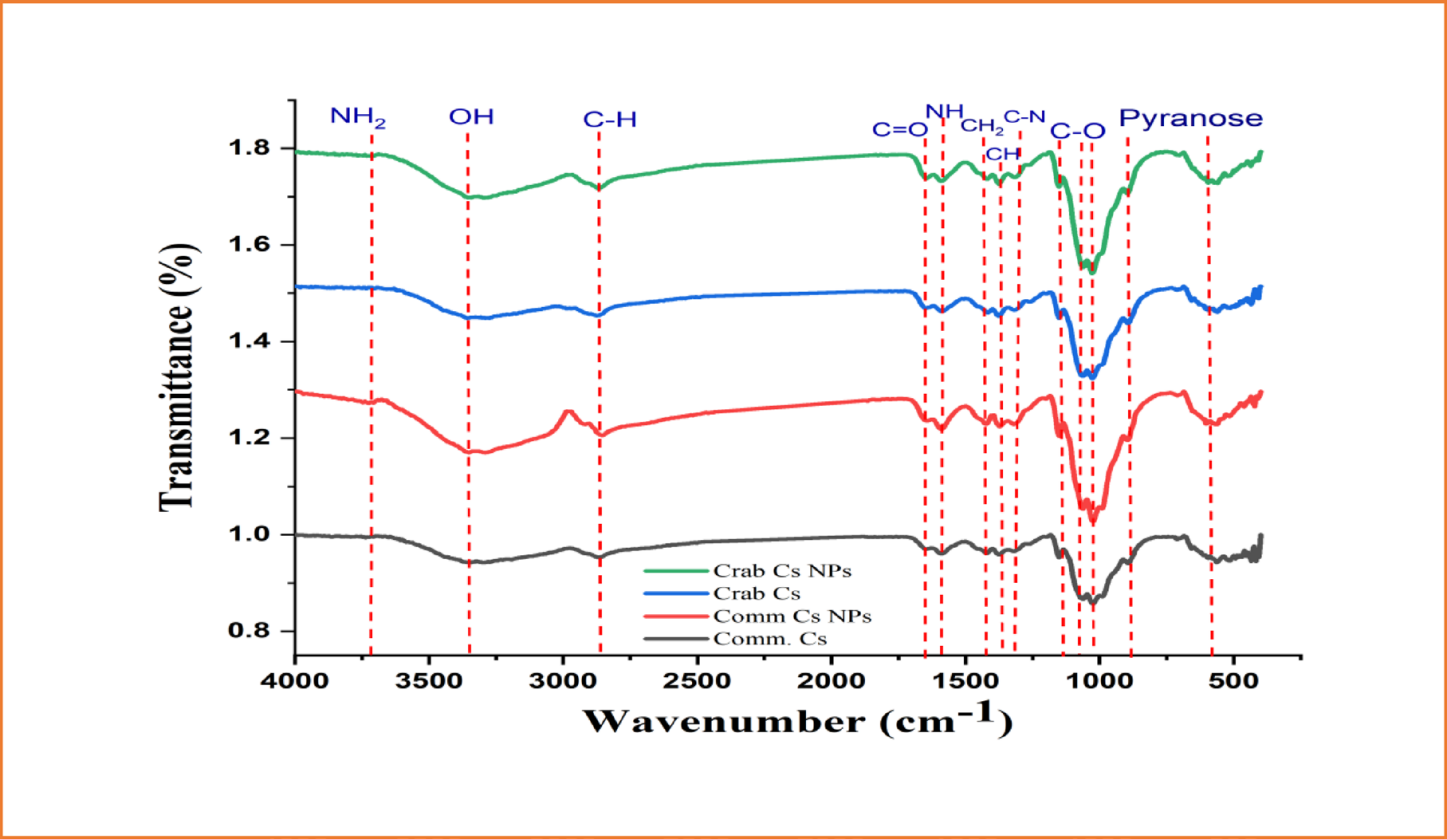
FTIR results of Crab CS, Crab CS NPs, Comm CS, and Comm CS NPs.

FTIR analysis is utilized to ascertain the biomolecules of the CS structure^52^. The spectral frequency ranging from 484 to 825 cm^1^ at all samples was attributed to the pyranose group. A notable feature of CS was the stretching of C–O–C and C–O bridges, observed in all samples that exceeded 1000 cm^1^(Wahab et al.)^8^. The bands at 1024.62, 1024.20, 1027.22, and 1027.63 cm^1^ presented the stretching vibration of C-O of primary alcohols; those at 1061.03, 1061.56, 1064.72, and 1061.27 cm^1^ might be indicative of the C-O stretching of secondary alcohols, while those at 1150.91, 1151.14, 1151.07, 1151.59 cm^1^ referred to the C-O stretching vibrations of tertiary alcohols for Crab CS and Crab CS NPs, Comm CS, and Comm CS NPs, respectively (Fig. 2).

The vibrational band of C-N appears at 1319.11, 1320.45, 1318.07 and 1316.82 cm^1^ for Crab CS, Crab CS NPs, Comm CS, and Comm CS NPs, respectively. The bands at 1375.63, 1372.14, 1375.34, and 1375.47 cm^1^ are credited to the C-H bending vibration of CH, while those at 1419.62, 1423.21, 1416.69, and 1419.29 cm^1^ referred to the C-H bending vibration of CH_2_ for Crab CS, Crab CS NPs, Comm CS, and Comm CS NPs, respectively. The signals around 1500 cm-1 at 1590.7, 1599.8, 1588.36, and 1591.05 cm^1^ referred to the C-N stretching vibrations of Crab CS, Crab CS NPs, Comm CS, and Comm CS NPs, respectively. Wahab et al.^8^ attributed the vibrational peaks around 1550, 1559, 1555, and 1557 cm^1^ to the amine III bands C–N stretch for comm CS, freshwater, marine, and brackish water CS crabs, respectively. The occurrence of peaks at 1643.81, 1650.8, 1651.65, and 1645.89 cm^1^ for crab CS, crab CS NPs, comm CS, and comm CS NPs referred to the C = O of acetamide groups of remaining chitin; however, the low intensity of these peaks referred to a good deacetylation of chitin extracted from crab.

The bands at 2870.05, 2855.04, 2874.34, and 2871.82 cm^1^ assigned the C-H aliphatic stretching group (CH and CH_2_). The appearance of peaks at 3294.15, 3351.47, 3257.93, and 3294.87 cm^1^ referred to the N-H stretching of the NH_2_ group.

The peaks at 3736.24, 3725.38, 3717, and 3717.04 cm^1^ assigned the O-H group (free from hydrogen bonding) for crab CS and crab CS NPs, comm CS, and comm CS NPs, respectively. The FTIR spectrum analysis indicated that the CS extracted from crab and its nanoparticles displayed a characteristic α-CS structure. The FTIR findings are equivalent to comm CS and previous documents in the literature data^44^. Previous works have validated the structural heterogeneity of CS contingent upon the duration required for the deacetylation process^53^.

DD was computed to be 98.79 and 97.21% of both crab CS and crab CS NPs, respectively. This finding was similar to the DD of 99.53 and 98.52% for both comm CS and comm CS NPs, respectively. The high DD values obtained in the present study may be attributed to the combined effect of concentrated NaOH solution (70%), elevated deacetylation temperature (100 °C), prolonged reaction time (5 h), and the optimized solid-to-liquid ratio (1:14 w/v). These conditions enhance the removal of acetyl groups from chitin and promote the formation of free amino groups in chitosan.

Similarly, Águila-Almanza et al.^52^ produced CS with a high DD of 70.8% by employing 50% NaOH, compared with 40% NaOH, which resulted in 64.5% DD. In Comparison, Nardo et al.^53^ produced CS mechanochemically with a degree of deacetylation (DD) of 23% by combining chitin and NaOH in a 1:5 (w/v) ratio using a ball mill for 30 to 90 min. Similar observations were reported by *Águila-Almanza et al.*^54^, who demonstrated that increasing NaOH concentration improved DD values, while Anusha et al.^54^ and He et al.^55^ reported that higher deacetylation temperatures and extended alkaline treatment enhanced chitosan deacetylation efficiency. An analogous study conducted by (Anusha et al.)^54^ utilized chitin extracted from squid pens to synthesize CS with the ball mill method in a nitrogen atmosphere. Following 2 h of ball milling, the resultant CS exhibited a DD of around 80%^56^. The DD results of the present work were higher than other previous studies that prove the excellent and promising capacity of 70% NaOH in the deacetylation process as well as the concentration ratio (1:14 w/v) and the deacetylation temperature (100 °C) and time (5 h). Despite the improvement in DD under strong alkaline conditions, excessive NaOH concentration, high temperature, and prolonged treatment may cause partial hydrolysis of glycosidic bonds and dissolution of low-molecular-weight fractions, thereby reducing the final chitosan yield. Consequently, chitosan extraction requires balancing deacetylation efficiency and polymer preservation to achieve both high DD and acceptable yield^25^.

#### XRD analysis

**Fig. 3:**
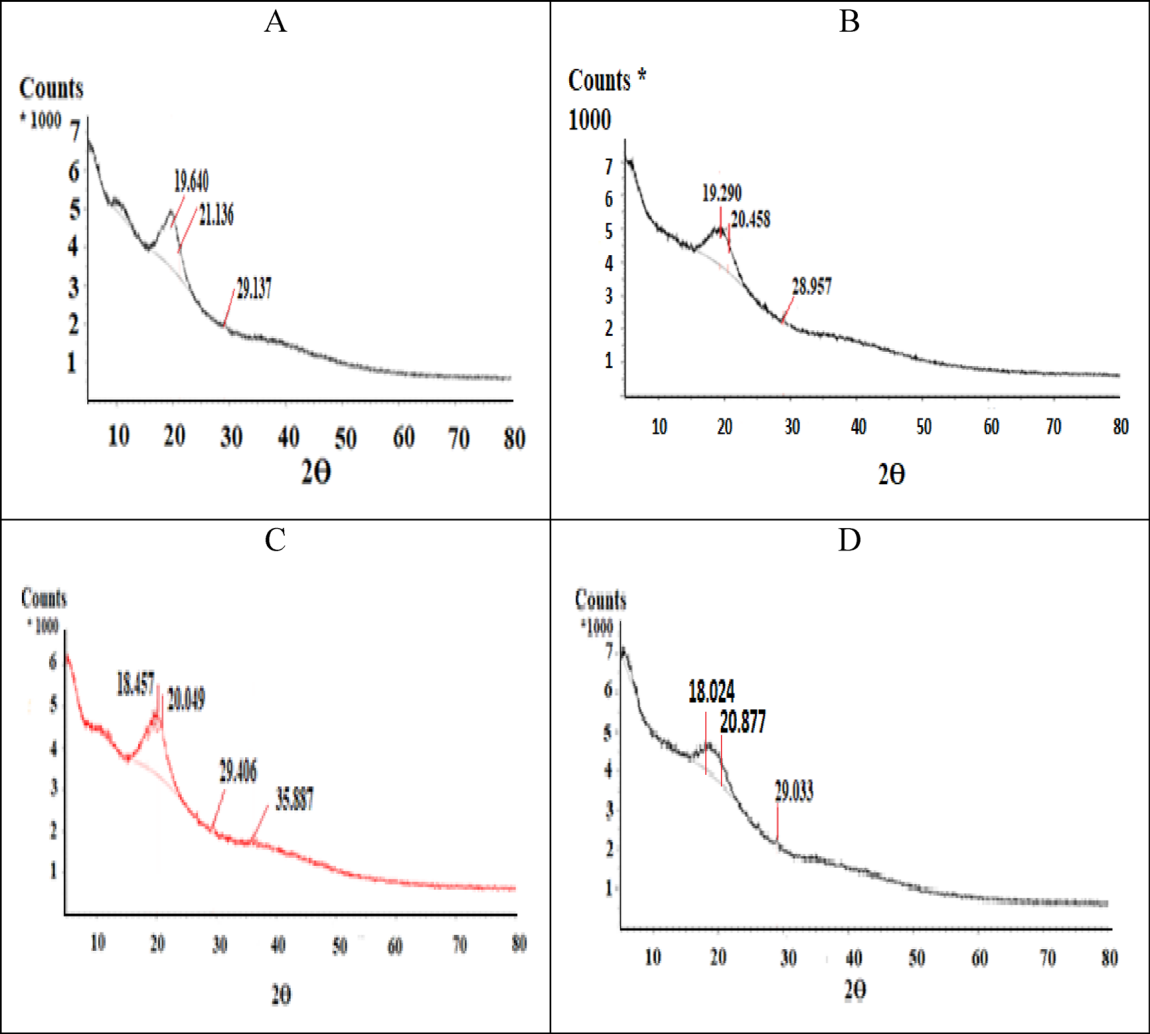
XRD of (**A**) Crab CS, (**B**) Crab CS NPs, (**C**) Comm CS and (**D**) Comm CS NPs.

The structure of CS and its NPs was investigated by the XRD diffractogram and compared with the commercial ones (Fig. 3). Crab CS showed two broad peaks at 19.64^◦^, 21.13^◦^ and one weak peak at 29.13º, while crab CS NPs showed their characteristic broad peaks at 19.299^◦^, 20.453^◦^, in addition to a weak peak at 28.957^◦^. The small shift in 2Ɵ measurements may be attributed to the difference in particle size between CS and its nanoparticle form. In a similar pattern, Comm. CS showed two broad diffraction peaks at 18.4^◦^, 20.05^◦^, and two weak peaks at 29.4^◦^, and 35.88^◦^ while Comm. CS NPs showed broad peaks at 18.4^◦^, 20.05^◦^, and a weak peak at 29.4^◦^. The characteristic diffraction signals at 2Ɵ ~ 20º, were recognized as the main diffraction signal in all samples, which assigned the hkl of (110) of amide II (NH_2_) and is indicative of the α-CS form^57^. As this characteristic peak is broad, XRD analysis investigated the amorphous structure of all CS samples. Thus, XRD analysis is a beneficial tool to differentiate between the crystalline and amorphous forms of CS. The peak observed at 2θ ~ 29º of all samples may correspond to the (130) lattice plane of the α-chitosan (α-CS), indicating that the three-dimensional configuration of crab chitin conforms to the α-polymorph, with the CS filaments arranged in an antiparallel orientation^4^. The α-CS strands display an α-orientation that they are coupled by intra-strand $C - OH \cdots OH - C$ hydrogen bonds, while the α-CS layers are further interconnected via progressive $C - OH \cdots OH - C$ hydrogen bonding connections^58^. Furthermore, the weak peaks of 2 Ɵ ~ 29º in all samples may be representative of high DD, aligning with the findings of (He et al.)^55^ who reported the peak intensity diminished as the DD value increased in ultra-high CS.

Crystallinity index (CrI) was estimated from XRD patterns for the prepared samples using Segal equation based on intensity of crystalline peak around 2θ ≈ 20 and on the amorphous region close to 2θ = 16° as follows:

4 $$\:CrI\left(\%\right)=\frac{({I}_{110\:\:}-\:{I}_{am})}{{I}_{110}}\times\:100$$

The CrI values calculated were 40.5% for the Crab CS, 31.9% for the Crab CS NPs, 52.9% for Comm CS and 45.7% for Comm CS NPs. CrI calculations derived from XRD diffractograms confirmed the predominantly amorphous nature of the synthesized chitosan samples. The broad diffraction peaks observed around 2θ ≈ 20° together with the relatively low CrI values indicate reduced molecular ordering within the polymer matrix. The results showed that commercial chitosan had a relatively more crystalline structure than samples from crab. Furthermore, the nanoparticle samples exhibited slightly lower crystallinity than the corresponding bulk chitosan, suggesting that the mechanical milling process contributed to disruption of intermolecular hydrogen bonding and crystalline domains.

Rostamabadi et al.^59^ attributed the low degree of crystallinity in biopolymers to the formation of inter- and intra-hydrogen bonds between the molecules. The peak at 2 Ɵ of 35.88^◦^ for comm. CS may be representative of the chitin residuals in comm. CS (Fig. 3). However, Meena et al.^60^ reported the crystalline form of CS to the formation of a strong peak at 25.4º, while Wang et al.^61^ distinguished the amorphous CS structure by the broad peaks noticeable around 25.4^◦^ and 38.0^◦^ in the XRD analysis. The absence of diffraction peaks at 10◦ referred to the absence of amide I (–N–CO–CH_3_) of residual chitin and hence reflected a good deacetylation process, this finding was in agreement with the results of Ma et al.^62^. However, the peaks below 20◦ (18–19^◦^) were due to the existence of few elements in isolated CS, which aligns with prior observations of crab biowaste^61^, and king crab^63^. Crab CS reflected slightly lower diffraction angles compared with those of comm CS, the outcomes matched with the results of CS derived from crab and squilla by^64^. On the other hand, Garenaux et al.^65^ attributed the amorphous form of extracted CS to the presence of remaining proteins, lipids, and minerals in the sample, which lowers the relative peak intensity. In comparison, Marei et al.^66^ observed that shrimp chitosan exhibited two significant diffraction peaks at 9.4° and 20.2°, beside two lesser peaks at 22.0° and 26.8°; conversely, locust chitosan revealed three peaks at 9.3°, 20.2°, and 24.4°. Furthermore, prior research on insects, shrimp, and crabs revealed analogous diffraction peaks within the range of 10° to 20°^67^. In a previous work^34^, extracted CS from some crab shells that recorded a higher diffraction peak at 25.42° for crab CS and 17.578° for its NPs form, respectively. The Debye-Scherrer equation elucidates the correlation between peak width in the spectrum of X-rays and particle size^68^. According to Debye-Scherrer calculations, the particle size was 84.2 nm for Crab CS NPs and 85.74 nm for Comm CS NPs. The Particle size calculated with the Debye–Scherrer equation reflects the mean crystallite size of nanoparticles and not their hydrodynamic diameter in suspension.

#### Scanning Electron Microscopy analysis (SEM)

**Fig. 4:**
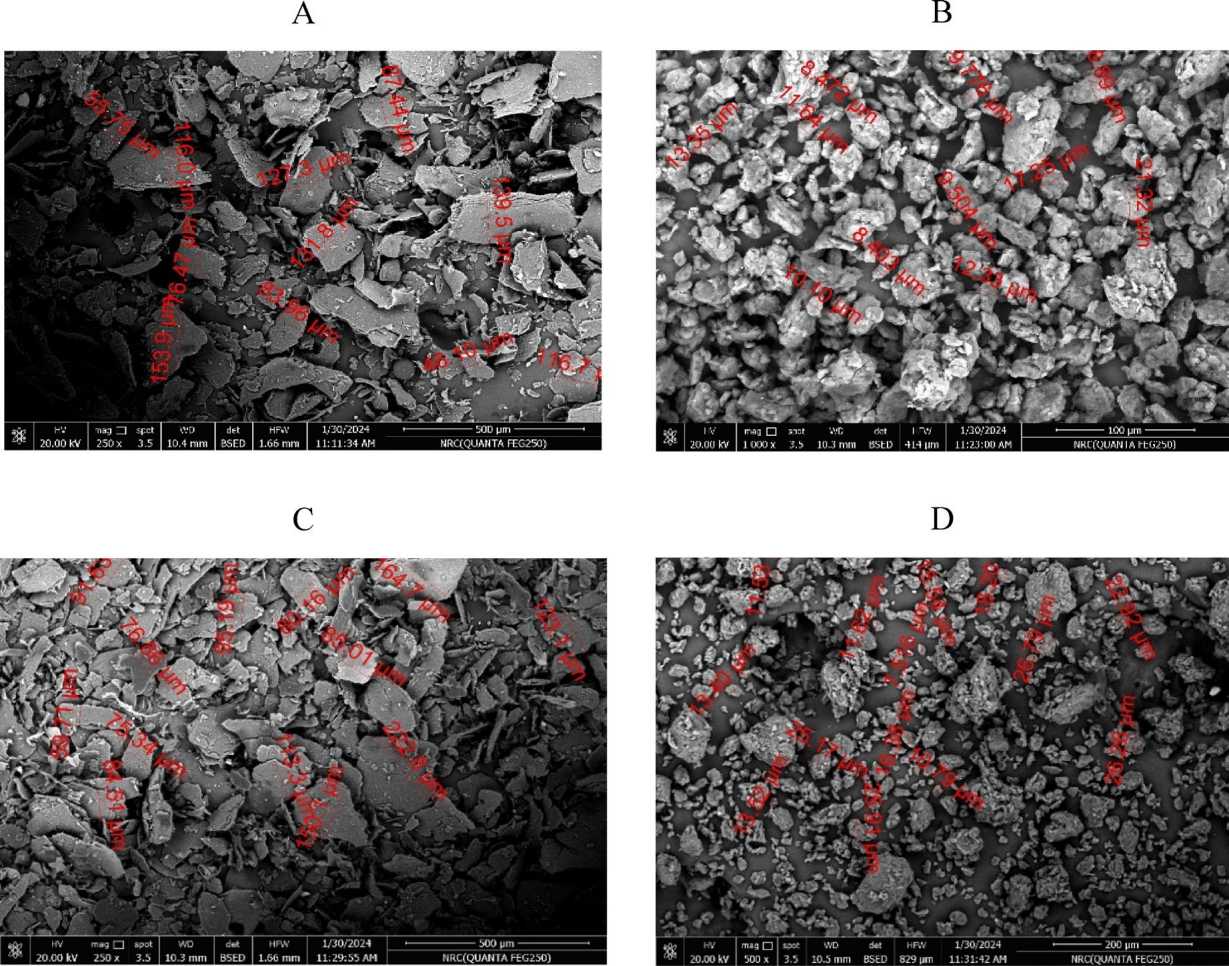
SEM Images of (**A**) Crab CS, (**B**) Crab CS NPs, (**C**) Comm CS and (**D**) Comm CS NPs.

SEM micrographs outlined a variation in the roughness and surface morphology among the different types of CS and its nanoparticle types. Marie et al.^66^ reported notable differences in the surface morphology of CS derived from a variety of sources, including insects, fungi, krill, and crabs exhibited. The overall morphology of CS at all samples exhibited an amorphous structure under SEM that aligned with the XRD results (Fig. 4). Moreover, crab CS showed a flake-like, hard, heterogenous, and rough surface with pores between 46.1 and 153.9 μm while crab CS NPs showed a hard surface with cubic to spherical shapes and pores between 8.4 and 21.32 μm with smaller sizes depending upon the particle size reduction using the ball milling technique.

In comparison, comm CS represents a combination of rough and smooth surface morphology with fibrous heterogenous structure and pores between 59.71 and 222.8 μm, while comm CS NPs showed more fine particles with spherical shape and pores between 7.5 and 26.79 μm. In fact, the surface morphology of CS is a crucial factor in its use across numerous applications. Chitosan, characterized by a porous surface structure, exhibits exceptional adsorption ability for metal ions, rendering it appropriate for metal ion uptake activities^14^. Conversely, chitosan with a fibrous surface morphology can be employed in the textiles sector^69^.

In comparison, Talab et al.^34,35^ reported a very smooth surface of CS and a porous-fibrous external structure of CS NPs extracted from shrimp shell waste. Talab et al.^34^ confirmed the differences in the CS surface texture depending mainly on its origin. Wahab et al.^8^ extracted Cs from Gecarcinucoidea that exhibited a more rigid and ridge-like shape devoid of cavities compared to its comm CS. Anand et al.^69^ extracted CS from *P. pelagicus* that exhibited a rough, loosed and sheet-like SEM morphology similar to those documented from mud crab and squilla species. Furthermore, the CS extracted from *Scylla sp.* exhibited a sponge-like surface morphology with few gaps, corresponding to that of the blue crab^70^ and the horseshoe crab^53^. In the same context, previous works reported porous surfaces and nanofiber structures of CS derived from crustaceans, including krill, *Gammarus argaeus*, and pink shrimp, by^29^. Nonetheless, holes have not been observed in certain chitosan samples derived from fungus^71^.

#### Elemental analysis (EDX)

**Fig. 5:**
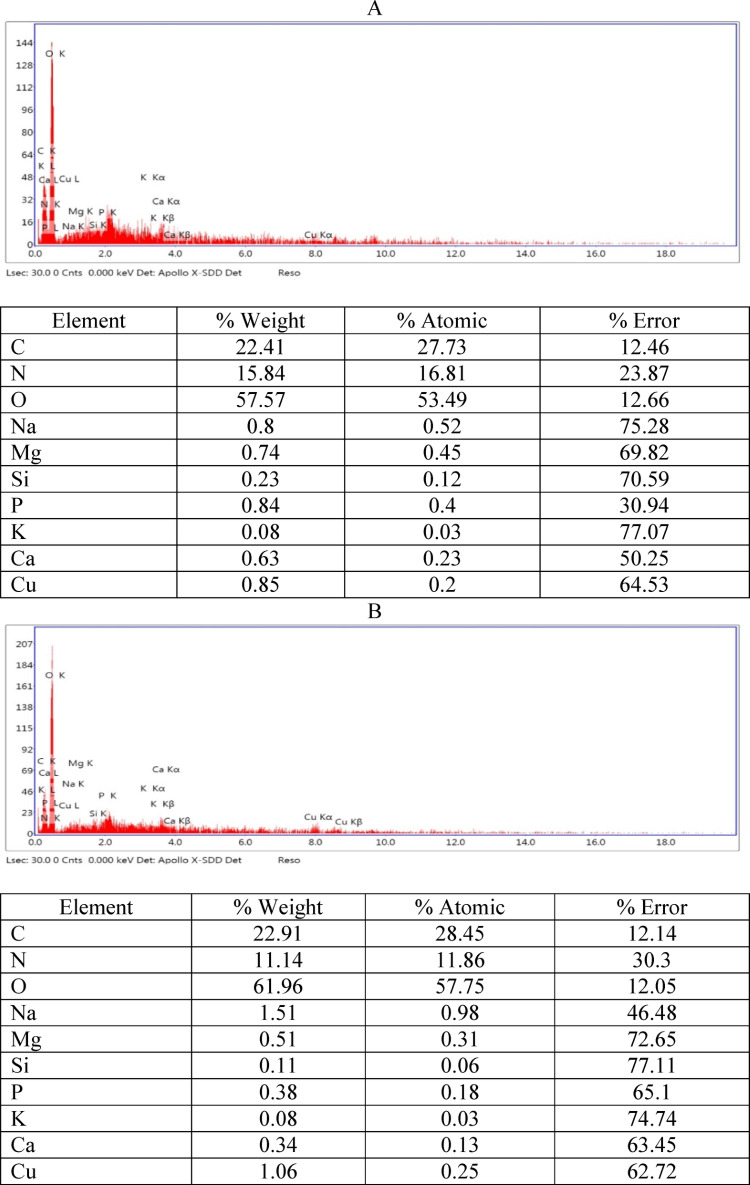

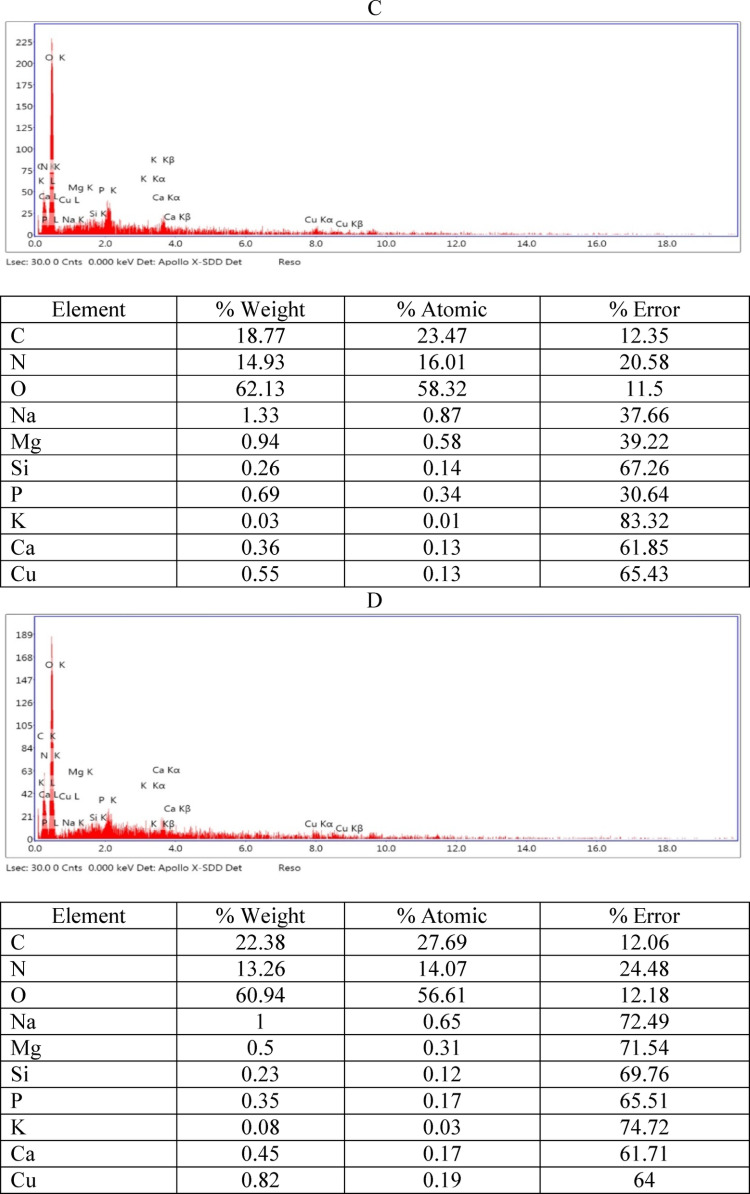
EDX Elemental Composition of (**A**) Crab CS, (**B**) Crab CS NPs, (**C**) Comm CS and (**D**) Comm CS NPs.

The elemental composition of extracted crab CS and its NPs was analyzed by EDX and compared with the commercial ones (Fig. 5). The EDX can ascertain the quantity and energy of X-rays generated from the material in order to detect the constituent elements^69^. This analysis can be employed to test the acid capacity of demineralization of chitin and hence the purity of the extracted CS and its nanoform. CS mainly comprises oxygen (O), carbon (C), and nitrogen (N) as its main elements.

The EDX results indicated the existence of C, N, and O with varied intensities depending on their source in all samples (Fig. 5). Crab CS mainly consisted of C (22.41%), N (15.84%), O (57.57%) by weight, indicating a high purity of chitosan exceeding 95.82% wt. However, other elements-phosphorus (P), calcium (Ca), potassium (K), sodium (Na), Magnesium (Mg), silicate (Si), and copper (Cu) were detected in trace percentages (~ 4.17% wt). In a similar pattern, crab CS NPs comprised C (22.91%), N (11.14%), O (61.96%) by weight, indicating a 96.01% of purity CS NPs. Upon the nanoparticle formation, the ratio between the different elements varied, where % O increased while % N decreased through the ball milling technique. The other elements of Na, Mg, Si, P, K, Ca, and C presented a total weight of 3.99% in the crab CS NPs. Accordingly, EDX is used to illustrate the purity of the chitin extraction and the deacetylation capacity.

The EDX results may prove the high capacity of the utilized acid to remove most elements from the crab exoskeletons. But some elements still exist in traces < 5% wt. This study referred to necessitating employing a more concentrated acid to eliminate these impurities. In comparison, comm. CS consists of C (18.77%), N (14.93%), and O (62.13%), with a total weight of 95.83% purity, with the existence of few elements of Na, Mg, Si, P, K, Ca, and Cu of about 4.16% wt. Moreover, crab CS NPs comprised C (22.38%), N (13.26%), and O (60.94%) with a total weight of 96.58% and some traces of minerals of approximately 3.43%. In the EDX mapping, the elemental composition of Na, Mg, Si, P, K, Ca, and Cu was 0.8, 0.74, 0.23, 0.84, 0.08, 0.63, 0.85% wt, respectively, of crab CS while was 1.51, 0.51, 0.11, 0.38, 0.08, 0.63, and 0.85% wt, respectively, of Crab CS NPs. With respect to commercial ones, the EDX elemental composition of Na, Mg, Si, P, K, Ca, and Cu was 1.33, 0.94, 0.26, 0.69, 0.03, 0.36, and 0.55% wt, respectively, of comm. CS while was 1, 0.5, 0.23, 0.35, 0.08, 0.45, and 0.82; respectively of Comm. CS NPs. In comparison, Talab et al.^34^ extracted CS and CS NPs from shrimp and crab exoskeletons in a previous work and reported that crab CS contained minerals of 3.09% Na, 0.22% Mg, 0.82%P, 0.21% K, 2.88% Ca, and 1.12% Cu that exhibited less purity than the current results. The residual trace minerals detected by EDX analysis may be attributed to incomplete demineralization during acid treatment, which can affect the applicability of prepared chitosan for very sensitive biomedical or food-grade applications. In this context, Talab et al.^72^ ascribed the inability of diluted acid to extract every trace element from shrimp and crab shells, in contrast to concentrated acids, which may only deposit little mineral residues.

#### Viscosity

**Fig. 6:**
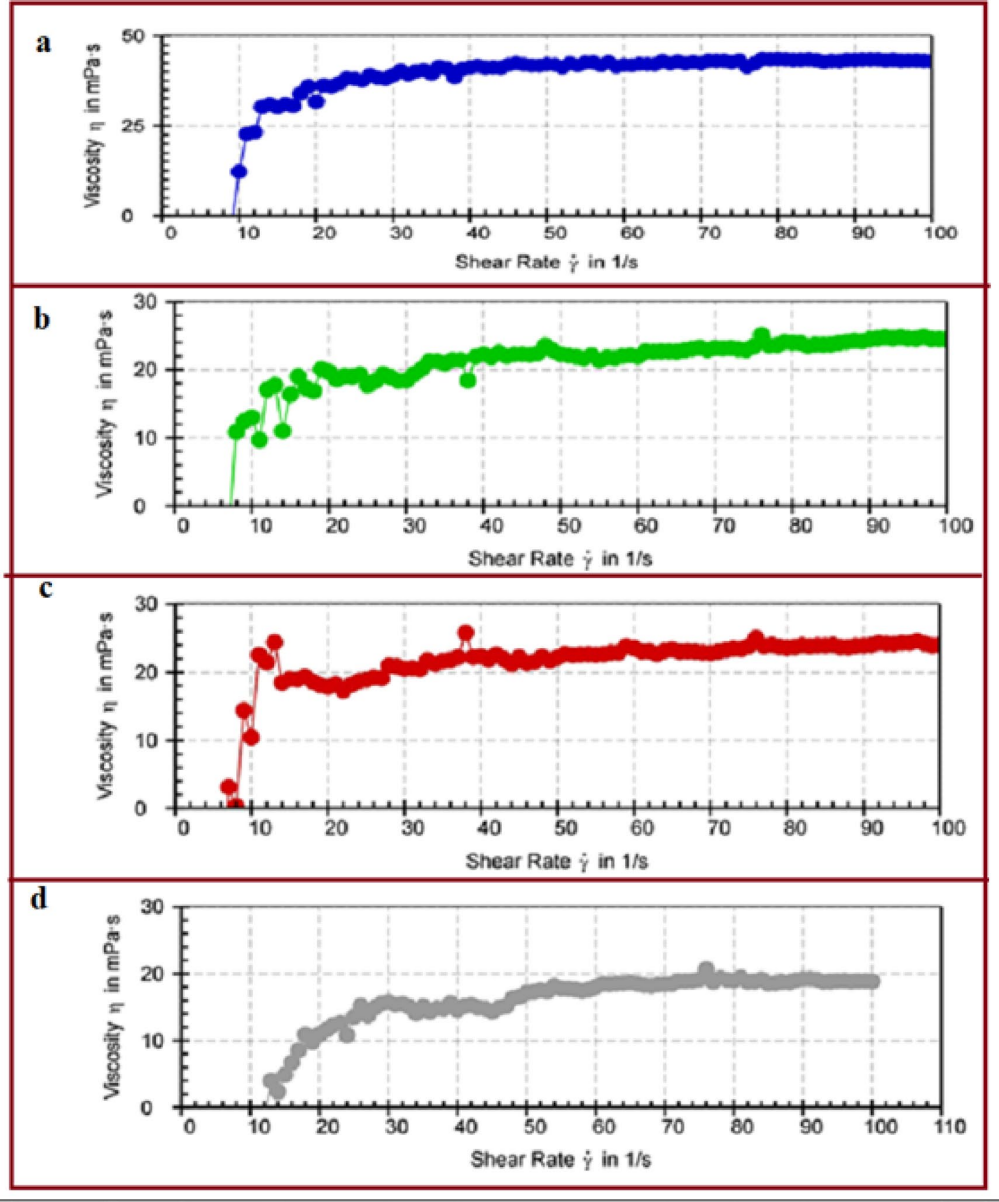
Viscosity Measurements of (**A**) Crab CS, (**B**) Crab CS NPs, (**C**) Comm CS and (**D**) Comm CS NPs.

The viscosity measurements for both Crab CS and Comm before and following the conversion to nanoparticles highlight significant distinctions in their rheological properties. Viscosity is fundamentally described as the proportion of shear stress to shear rate, typically demonstrating a decreasing or stabilizing pattern with increasing shear rate, which is indicative of shear-thinning behavior commonly observed in polymeric solutions. Crab CS starts with a higher viscosity (31.707 to 42.849 mPa·s), exhibiting irregular fluctuations that may stem from structural variances or aggregation phenomena. After undergoing nanoparticle conversion, its viscosity stabilizes within a lower range (19.659 to 24.498 mPa·s), (Fig. 6). In the case of Comm CS, the viscosity begins at 17.818 mPa·s and progressively rises to 24.024 mPa·s as the shear rate increases. However, post-nanoparticle conversion, the viscosity declined (10.942 to 18.864 mPa·s), implying that the formation of nanoparticles diminishes intermolecular interactions and overall flow resistance. This finding mirrors the behavior observed in crab CS NPs. A comparative analysis reveals that crab CS possesses a markedly higher viscosity than its commercial counterpart, suggesting a more complex molecular entanglement. Nonetheless, both CS types experience a reduction in viscosity following nanoparticle formation, thereby affirming that the presence of nano-sized particles enhances fluidity and mitigates macromolecular interactions. The viscosity of nanofluids is influenced by a multitude of elements, encompassing the base fluid, particle dimensions, quantity, morphology, dispersion technique, pH, temperature, agglomeration, and the Brownian motion of nanoparticles^73^. Among these, the size of the dispersed nanoparticles is a critical determinant of nanofluid viscosity. Investigations into the effects of particle dimensions on the viscous properties of nanofluids have yielded mixed results; some studies indicate that smaller particle sizes correlate with increased viscosity, while others suggest the opposite trend^74^.

Crab CS and its NPs showed high viscosity measurements and hence higher relatively molecular weight compared with comm CS and its NPs. Molecular weight is an essential factor that can extensively impact the physicochemical and rheological properties of chitosan and hence its applications in different sectors^75^.

Nanoparticle formation through mechanical milling decreased the apparent molecular weight owing to partial chain scission and depolymerization of the chitosan chains. The decrease in viscosity after the preparation of nanoparticles is consistent with decreased hydrodynamic volume and polymer chain length. In addition, the narrow particle size distribution obtained from nanoparticle synthesis as well as its contribution to dispersion stability and lower apparent polydispersity”. Zhang et al.^76^ examined the antimicrobial and antimutagenic properties of six different molecular weights of CS and realized that the antimicrobial properties enhanced as the molecular weight decreased. As lower molecular weights exhibit greater susceptibility to interactions with free radicals. This study extracted crab CS with higher viscosity measurements, and molecular weight, reflecting lower water solubility and biodegradability that in turn decreased its application in antimicrobial properties and even biomedical applications but may be useful for textile industries and wastewater treatment^77–79^.

#### Transmission Electron Microscopy (TEM)

**Fig. 7:**
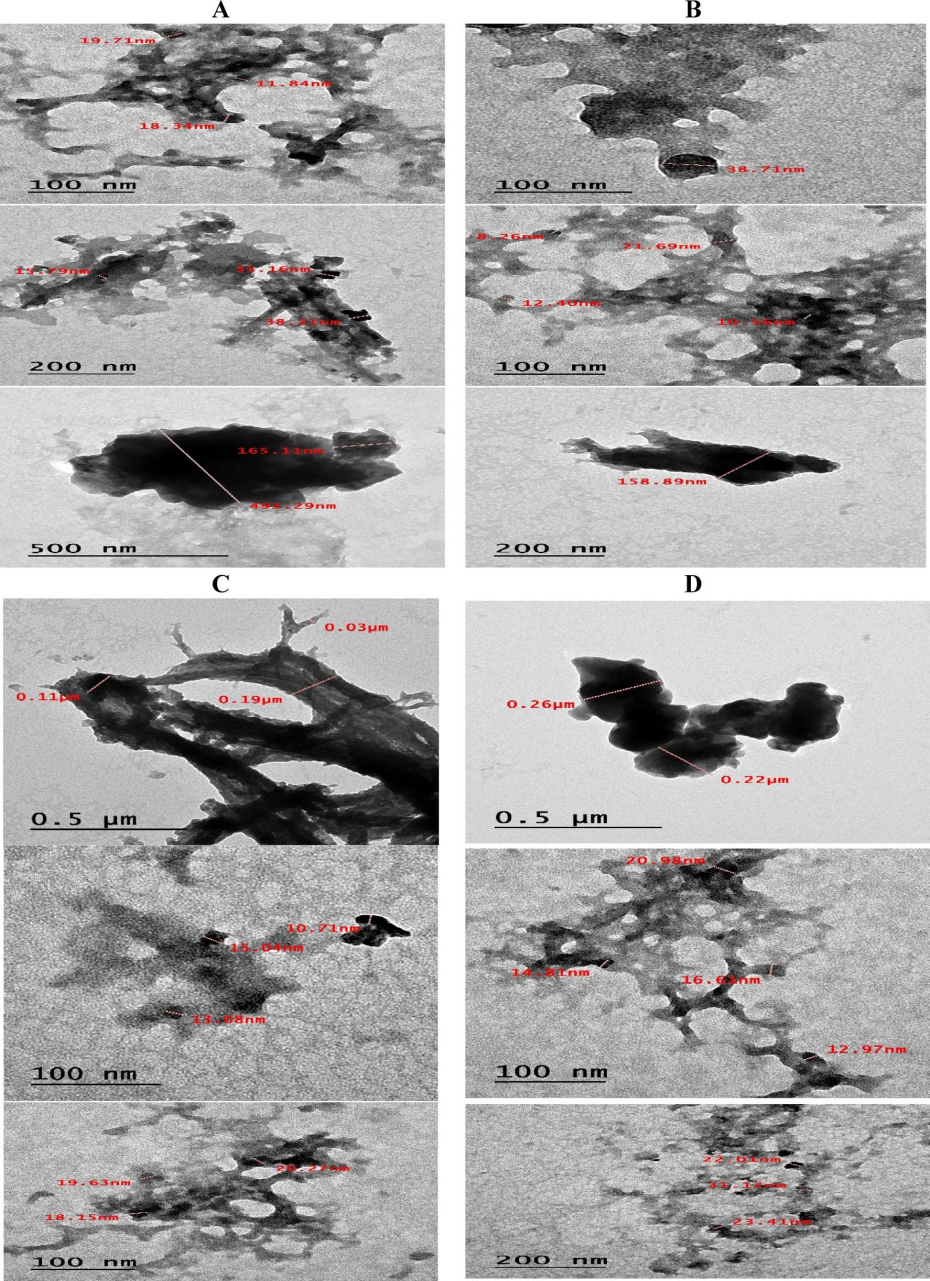
TEM of (**A**) Crab CS, (**B**) Crab CS NPs, (**C**) Comm CS and (**D**) Comm CS NPs.

The transmission electron micrograph (TEM) of chitosan and its nanoparticles are represented in (Fig. 7a-d). The micrograph depicts very smooth and fine surfaces which could contribute to its efficiency when applied industrially in a food industries, drug delivery system, etc. Similar micrographs and shapes were reported by^80^. The chitosan captured in this work demonstrated desirable physicochemical properties, but no application-oriented studies such as adsorption efficiency, antimicrobial activity, cytotoxicity or biocompatibility were performed in this study.

## Conclusion

The production of chitosan and chitosan nanoparticles from blue crab leftovers and the comparison of the results with commercial types were the work’s highlights. In contrast to commercial nanoparicles, which had a particle size of 245.47 ± 3.21 nm, zeta potential of 32.24 ± 0.72 mV, and smallest PDI of 0.191 ± 0.055, the DLS results of crab chitosan nanoparicles seemed to be the best, with minimum particle size of 192.74 ± 3.25 nm, zeta potential of 25.15 ± 1.19 mV, and smallest PDI of 0.228 ± 0.011. According to the FTIR and XRD investigations, α-chitosan mostly developed an amorphous structure with a higher degree of deacetylation. This study also recommended that extraction techniques be given more consideration in order to produce nanoparticles with logical designs.

## Data Availability

No datasets were generated or analysed during the current study.
